# Improved spatial learning and memory by perilla diet is correlated with immunoreactivities to neurofilament and α-synuclein in hilus of dentate gyrus

**DOI:** 10.1186/1477-5956-10-72

**Published:** 2012-12-05

**Authors:** Jinwoo Lee, Sunmin Park, Ju-Young Lee, Yeong Keun Yeo, Jong Sang Kim, Jinkyu Lim

**Affiliations:** 1 Major in Food Biomaterials, Kyungpook National University, Daegu, 702-701, South Korea

**Keywords:** Perilla, n-3 fatty acid, Cognition, Hippocampus, Proteome, 2-D gel, Immunohistochemistry

## Abstract

**Background:**

Perilla (*Perilla frutescens*) oil is very rich in α-linolenic acid, an omega-3 fatty acid. As it is widely reported that omega-3 fatty acid supplementation improves cognitive function in children and adults, feeding rats with perilla diets followed by analysis of proteomic changes in the hippocampus can provide valuable information on the mechanism of learning and memory at the molecular level. To identify proteins playing roles in learning and memory, differentially expressed proteins in the hippocampus of the 5 week old rats fed perilla diets for 3 weeks or 3 months were identified by proteomic analysis and validated by immunological assays.

**Results:**

The perilla diet groups showed improved spatial learning and memory performances in a T-maze test. They also displayed elevated level of 22:6n-3 fatty acid, an omega-3 fatty acid (*p*<0.05), in the brain compared to the control diet group. Quantitative proteomic analysis using 2-D gels as well as functional annotation grouping with the differentially expressed proteins in the hippocampus showed that those proteins involved in cytoskeleton and transport were the major differentially expressed proteins in the 3-week group, whereas those involved in energy metabolism, neuron projection and apoptosis in addition to cytoskeleton and transport were the major ones in the 3 month group. Differential protein expression in the hippocampus was validated by Western blotting using four selected proteins, known to be involved in synaptic plasticity; AMPA receptor, neurofilament, α-synuclein, and β-soluble NSF attachment protein. Brain sections from the perilla-diet groups showed enhanced immunoreactivities to α-synuclein and neurofilament. Especially, neurofilament immunoreactive cells manifested longer neurite projections in the hilus of dentate gyrus of the perilla-diet groups.

**Conclusion:**

Improved cognitive function upon administration of n-3 fatty acid-rich perilla diet is associated with the differential expression of hippocampal proteins related to cytoskeleton, energy metabolism, transport, neuro-projection, and apoptosis. Particularly, the enhanced immunoreactivities to α-synuclein and neurofilament in the hilus of dentate gyrus suggest that perilla diet supplementation promotes neuronal signaling and alters synaptic plasticity for improved learning and memory.

## Background

Memory is the process of storing information from learning, which acquires information about experiences over time. For long term memory generation, protein synthesis is required in the brain [1]. Further transient multiple protein expression as well as constitutive prolonged local increase in protein synthesis in the brain are influenced by learning [2]. Newly synthesized proteins are involved in the growth of new synapses, including both pre- and post-synaptic alterations [3]. As long term memory and synaptic plasticity require new protein synthesis, it has been reported that long term changes in neural circuits as well as long term modification of behavior can be regulated by translational control [2,4].

Many reports have shown that foods can affect the cognitive processes in the central nervous system, such as, cognitive impairment by Western diet consumption [5], potential efficacy of cognitive and psychological functioning by vitamins [6], protection of vulnerable neurons by flavonoids from fruits and berries [7], reducing the chances of developing cognitive impairment by omega-3 polyunsaturated fatty acid (n-3 PUFA) [8], *etc*.

It has been known that dietary omega-3 fatty acids improve memory and learning processes, and also affect gene expression in the brain [9-12]. Previous studies have shown that PUFA-enriched diets significantly alter the mRNA expression levels of several genes in central nervous tissue, and these effects might be related to the (n-3)/(n-6) balance of polyenoic fatty acids in the cell membrane. Modern diets enriched in saturated fatty acids and simple carbohydrates are often deficient in omega-3 fatty acids, which are essential fatty acids and must be obtained through dietary sources.

Perilla (*P*. *frutescens*) is a good source of omega-3 fatty acids (over 50% total fatty acid) [13] which are essential fatty acids that can be converted to docosahexanoic acid (DHA) in the liver and the developing brain, and contains one of the highest contents of omega-3 fatty acid among edible plant seeds [14].

In this study, 5 week old rats fed with either a perilla oil- or corn oil-based diet for 3 weeks or 3 months, after which the cognitive functions of rats and protein profiles in the hippocampus were determined. The brain proteome of rats with improved spatial cognition induced by perilla-diet intake was compared with that of rats fed with a regular diet. Here, we report the significantly altered expression of cognition-associated proteins in the brain upon perilla diet-intake. Differential expression of the proteins was confirmed by Western blotting analysis and immunohistochemistry. Our findings indicate that n-3 fatty acid supplementation could alter synaptic plasticity in learning and memory performance by generating new hippocampal neural membrane structures as well as by inducing specific protein expression.

## Results

### Improvement of spatial learning and memory by perilla diets

**Figure 1:**
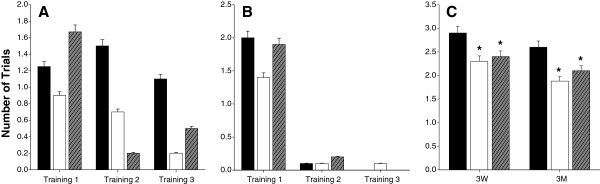
**Performances in a T-maze test.** Ten rats in each group fed control diet (black bar), perilla seed diet (white bar), or perilla oil diet (gray bar) for 3 weeks (**A**) and 3 months (**B**) were trained until they reach the goal box 6 times consecutively and the numbers of errors they made were counted in each training section. Likewise, after the three training sessions for two weeks, actual tests were given with a reversed goal box at the T-maze (**C**). The number of trials before reaching the goal box 6 consecutive times were significantly reduced (*, p<0.05) in rats fed perilla or perilla oil diet.

After 3 weeks or 3 months of perilla- or perilla oil-diet intake, 10 rats in each group were tested for learning and memory performance by T-maze tests. Throughout the training period, rats fed a control diet took a longer time to reach the goal box than the perilla-diet groups (Figure 1). Further, reversal of the goal box arm induced larger time difference between the perilla-diet groups and control group (data not shown). The number of trials it took to successfully reach the goal box five consecutive times was significantly altered by reversal of the goal box position. Rats fed with a control diet for 3 weeks took an average of 2.9 ± 0.32 trials, whereas rats fed perilla- or perilla oil-diet showed improved cognitive function (2.3 ± 0.48 or 2.4 ± 0.52 trials, respectively). (Figure 1). When these results were statistically compared with each other in a two-tailed *t*-test, the perilla-diet and perilla oil-diet groups showed significant differences compared to the control group with *p*-values of 0.0048 and 0.0192, respectively.

Along the same line, rats fed perilla- or perilla oil-diet for 3 months also showed significant improvements in spatial cognitive function (1.8 ± 0.42 or 2.1 ± 0.32 trials, respectively), whereas the control diet group took 2.6 ± 0.52 trials. These data show significant differences between the control group and perilla- and perilla oil-diet groups with *p*-values of 0.0014 and 0.0197, respectively.

### Fatty acid composition in the brain

**Figure 2:**
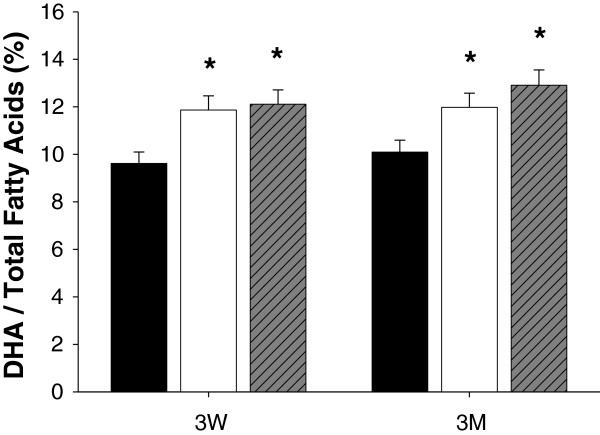
**Relative DHA content in the brain of rats.** Fatty acid contents in the brain of rats fed control diet (black bar), perilla diet (white bar) or perilla oil diet (grey bar) were analyzed by gas chromatography using an external fatty acid standard, heptadecanoic acid (17:0). The relative ratios of DHA in the total fatty acid were significantly increased in the brain of rats fed perilla or perilla oil diet (*, p<0.05).

Fatty acid composition in the brain of rats was analyzed by gas chromatography and normalized by comparison with an external standard, heptadecanoic acid (17:0). The results were compared between the control diet and perilla-diet groups (Additional file 1). The average percentage of 22:6n-3 fatty acids in the brain of the control group was 9.94±0.49, and this number increased to 12.10±0.60 or 12.43±0.62 in the perilla- or perilla oil diet groups, respectively, after 3 weeks (Figure 2). Similar differences were also observed in the 3 month feeding groups: 10.09±0.50 in the control group, 11.98±0.59 in the perilla group and 12.91±0.64 in the perilla oil group (Figure 2).

Statistical analysis of the results showed significant increase in 22:6n-3 fatty acids in the brain of perilla-diet fed rats after both 3 weeks and 3 months. Similar results were reported for rats fed n-3 fatty acids or perilla oil [10,12]. However, in our study, 18:3 fatty acid, which is the major fatty acid component of perilla, could not be detected in either the control or perilla-diet groups. Based on these results, we concluded that perilla-diet changed the fatty acid composition in the brain, consequently improving cognitive functions by altering the protein profile.

### Proteome profiles on 2-D gel and image analysis

Proteins extracted from pooled hippocampal tissues of rats from each group were separated on 2-D gels in order to quantitatively compare individual protein expression levels. Differentially expressed proteins compared to control group were identified by peptide mass fingerprinting and are listed in Tables 1 and 2. Among the 27 proteins selected from the 3 week groups, 33.3% of proteins (nine proteins) were clustered with cytoskeleton function with an enrichment score of 2.97 and *p*-value of 9.2 x 10^-4^, whereas 26% (seven proteins) were clustered with membrane bound vesicle or vesicle mediated transport with an enrichment score of 1.79 and p-value of 9.90 x 10^-4^. The remaining proteins were clustered with insufficient statistical significance (*p*>0.05) (Table 3), suggesting that the short period (3 weeks) of perilla-diet intake improved spatial cognition by changing cytoskeletal structure and vesicular transport in the hippocampus of rats. Functional annotation clustering of the 26 proteins selected from the 3 month perilla-diet intake groups resulted in seven annotation groups with enrichment scores higher than 2.1 (*p*<0.0013) (Table 3). Among the seven annotation clusters, the biological functions of the proteins could be separated into five clusters: energy metabolism, vesicle-mediated transport, cytoskeleton, neuron projection, and regulation of apoptosis. In addition to the annotation clusters of the proteins from the brains of the 3 week group, proteins from the brains of the 3 month groups could be separated into more clusters, including energy metabolism (27%), neuron projection (27%), and regulation of apoptosis (27%), suggesting that more diverse mechanisms of learning and memory are required as the period of perilla-diet treatment increases and the rats become older. To test whether or not annotation clusters from the differentially expressed proteins are a simple representation of the total proteins content in the brain, we randomly picked 20 abundant proteins (Additional file 2) from the hippocampal proteins separated by 2-D gel electrophoresis and identified them for annotation clustering (Table 3). Interestingly, we found that the functions of major proteins in the brain could be grouped into several clusters. The three enriched clusters in the brain, energy metabolism, neurogenesis and cytoskeletons, are also the major functional groups in the brain but with lower percentages and higher *p*-values than the annotation clusters of brain proteins from rats fed perilla-diets. This suggests that the annotation clusters of the differentially expressed proteins in the hippocampus are not simple representations of the major protein groups in the brain.

**Table 1:** Differentially expressed proteins in the hippocampus of rats fed perilla diets for 3 weeks

**Accession number**	**MOWSE score**	**Coverage (%)**	**Protein name**	**log(P/C)**^**a**^	**log(O/C)**^**b**^
P11598	1.38E+08	22.8	Protein disulfide-isomerase A3 precursor	**+**^**c**^	**+**
O35796	1650	16.8	Complement component 1Q-binding protein	**+**	**0**^**d**^
P28480	369942	23.7	T-complex protein1 subunit alpha	**+**	**+**
P63102	277629	26.1	14-3-3 protein zeta/delta	**0**	**+**
Q5U2Z5	770605	18.2	Uncharacterized protein KIAA0082 homolog	**0**	**+**
P02770	9.97E+19	45.7	Serum albumin precursor	**-**^**e**^	**-**
P85834	4.31E+06	30.5	Elongation factor Tu, mitochondrial precursor	**-**	**-**
P50399	673570	34.4	Rab GDP dissociation inhibitor beta	**+**	**+**
Q99NA5	14811	13.7	Isocitrate dehydrogenase NAD] subunit alpha	**0**	**+**
Q9Z214	22779	25.1	Homer protein homolog 1	**0**	**+**
Q6P9V9	50742	17.5	Tubulin alpha-1B chain	**0**	**+**
P11598	1.65E+07	21	Protein disulfide-isomerase A3 precursor	**-**	**+**
Q4V8G7	14578	16.6	Centromere protein U	**-**	**-**
P12839	2.10E+08	28	Neurofilament medium polypeptide	**-**	**-**
Q6QLM7	67576	10.2	Kinesin heavy chain isoform 5A	**-**	**-**
P54921	7.91E+15	66.4	Alpha-soluble NSF attachment protein	**-**	**-**
P37377	2069	37.9	Alpha-synuclein	**+**	**+**
P12075	2334	21.7	Cytochrome c oxidase subunit 5B	**+**	**0**
P05370	105594	20	Glucose-6-phosphate1-dehydrogenase	**+**	**+**
P63018 M	1.08E+08	22.3	Heat shock cognate 71 kDa protein	**+**	**+**
P85969	1.42E+07	25.3	Beta-soluble NSF attachment protein	**-**	**-**
Q99JY9 M	2.68E+09	32.5	Actin-related protein 3	**-**	**-**
P62815 M	1.22E+11	27	V-type proton ATPase subunit B, brain isoform	**-**	**+**
Q9R0Q7 M	230700	45	Prostaglandin E synthase 3	**+**	**+**
P36876	2501	14.1	Serine/threonine-protein phosphatase 2A	**-**	**+**
Q6PEC4 M	6321	27	S-phase kinase-associated protein 1	**-**	**+**
Q9WTT6	2.65E+07	24.4	Guanine deaminase	**-**	**-**

a, log value of the ratio of the spot intensity of perilla diet group (P) over control group (C).

b, log value of the ratio of the spot intensity of perilla oil diet group (O) over control group (C).

c, + means that the ratio of P/C or O/C over 1.2 is recognized as increased expression, and the value of log(P/C) or log(O/C) is over 0.079.

d, 0 means that the ratio of P/C or O/C between 0.8 and 1.2 are recognized as unchanged, and the value of log(P/C) or log(O/C) is 0.

e, - means that the ratio of P/C or O/C lower than 0.8 recognized as decreased expression, and the log values are under −0.097.

**Table 2:** **Differentially expressed proteins in the hippocampus of rats fed perilla**-**diets for 3 months**

**Accession number**	**MOWSE score**	**Coverage (%)**	**Identified protein**	**log(P/C)**	**log(O/C)**
P62260	2.17E+12	67.1	14-3-3 protein epsilon	**+**	**+**
P63018	3.93E+06	19.8	Heat shock cognate 71 kDa protein	**+**	**+**
P37377	2069	37.9	Alpha-synuclein	**+**	**+**
P07323	1.38E+14	55.1	Gamma-enolase	**-**	**-**
P10719	1.14E+13	33.3	ATP synthase subunit beta, mitochondrial	**-**	**-**
P23565	1.30E+13	42.6	Alpha-internexin	**+**	**+**
P06761	1.82E+16	39.1	78 kDa glucose-regulated protein	**-**	**-**
P12839	4.40E+08	22.5	Neurofilament medium polypeptide	**+**	**+**
P85969	1.84E+13	59.9	Beta-soluble NSF attachment protein	**+**	**+**
P63018	2.91E+07	22	Heat shock cognate 71 kDa protein	**+**	**0**
P46462	8.33E+16	40.3	Transitional endoplasmic reticulum ATPase	**0**	**+**
P70566	3.88E+06	31.9	Tropomodulin-2	**-**	**-**
P63004	8881	21	Platelet-activating factor acetylhydrolase IB subunit beta	**-**	**0**
O88989	743497	27.2	Malate dehydrogenase, cytoplasmic	**-**	**0**
P61158	1.10E+07	24.4	Actin-related protein 3	**-**	**-**
P04764	2.91E+09	39.6	Alpha-enolase	**0**	**+**
Q05982	4.87E+07	65.1	Nucleoside diphosphate kinase A	**-**	**-**
P11598	8.32E+10	33.9	Protein disulfide-isomerase A3	**-**	**0**
P02770	1.07E+10	30.3	Serum albumin	**-**	**+**
P27605	101710	34.9	Hypoxanthine-guanine phosphoribosyltransferase	**-**	**+**
Q9JJM9	4.31E+09	36.6	Septin-5	**-**	**+**
P47942	8.82E+08	28.8	Dihydropyrimidinase-related protein 2	**+**	**+**
P53534	1.34E+11	27.1	Glycogen phosphorylase, brain form	**0**	**+**
P05708	1.79E+14	24.8	Hexokinase-1	**0**	**+**
P21575	2.18E+18	37.8	Dynamin-1	**0**	**+**
Q9QUL6	1.49E+10	20.6	Vesicle-fusing ATPase	**-**	**0**

**Table 3:** **Functional annotation clustering of the proteins differentially expressed in the hippocampus of rats fed perilla**-**diets**

**Annotation cluster**	**Number of proteins**	**Enrichment score**	**p-value**
**3 week-feeding**			
cytoskeleton	9	2.97	9.20E-04
membrane-bounded vesicle	7	1.79	9.90E-04
vesicle-mediated transport	7	1.4	1.70E-04
**3 month-feeding**			
vesicle-mediated transport	8	2.28	1.20E-05
cytoskeleton	9	2.24	4.90E-04
neuron projection	7	2.17	2.40E-04
regulation of apoptosis	7	2.1	1.30E-03
**20 major proteins**			
neurofilament cytoskeleton organization	3	3.32	6.70E-05
glycolysis	3	1.83	1.80E-03
regulation of axonogenesis	3	1.6	4.00E-03

### Western blot analysis

To verify the results of differential protein expressions in the brain upon perilla-diet intake, Western blotting analyses using antibodies against the four selected proteins were performed. Selection of protein was carried out according to the availability and specificity of the antibodies, reproducibility and fold differences of the proteomic results, and involvement of the proteins in synaptic plasticity.

**Figure 3:**
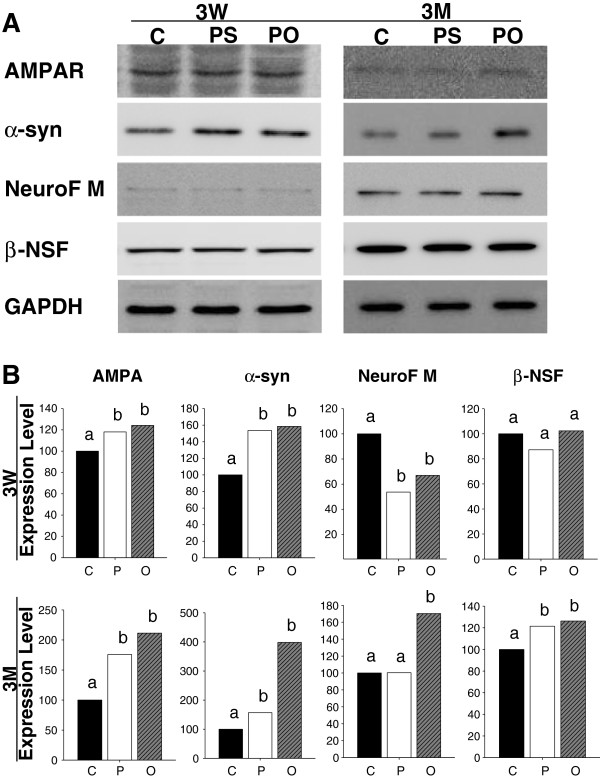
**Western blotting analysis on the hippocampus of rats fed perilla diets for 3 weeks (3W) and 3 months (3M).A**. The bands of hippocampal proteins separated on SDS-PAGE and transferred to PVDF membrane were incubated with antibodies against AMPA receptor (AMPAR), α-synuclein (α-syn), neurofilament M (NeuroF M), and β-soluble NSF attachment protein (β-NSF) and decorated with a chemiluminescence. **B**. The intensities of the bands were captured with a densitometer and normalized according to that of GAPDH. The normalized band intensities were compared between control (C) and perilla (P) or perilla oil (O) diet groups, and presented as relative expressions (%) with the control group as 100%. Letters a and b on the bar graphs designate differences between the expression levels in each group with different letters for significant differences (p <0.05).

Since AMPA receptor subunits are essential for hippocampal synaptic plasticity, spatial learning and memory, as well as long term potentiation induction and maintenance [15-17], an antibody against AMPA receptor was used to investigate improved spatial learning and memory in rats supplemented with perilla-diets. After 3 weeks of perilla-diet intake, there was a significant increase in AMPA receptor expression, a positive control for learning and memory improvement, in the hippocampus (Figure 3). AMPA receptor protein was also differentially expressed in the hippocampus of rats supplemented with perilla-diets for 3 months. α-synuclein protein showed a good correlation with the proteomic data from Western blotting analysis. Protein expression in the hippocampus significantly increased upon both perilla- and perilla oil-diet supplementation for 3 weeks or 3 months. Neurofilament was down-regulated in both the perilla-diet and perilla oil-diet groups after 3 weeks, but it was up-regulated in the 3 month perilla oil-diet group. β-Soluble NSF protein showed slight down-regulation in the 3 week samples of perilla-diet group but up-regulation in the 3 month samples of both perilla- and perilla oil-diet groups. Overall, the fold changes of expression level in AMPA receptor, α-synuclein, neurofilament, and β-soluble NSF protein were correlated with the proteomic data and were always greater in the perilla oil-diet group compared to perilla-diet group in Western blotting analysis.

### Improved spatial learning and memory are associated with increased immunoreactivity to α-synuclein and neurofilament in dentate gyrus

**Figure 4:**
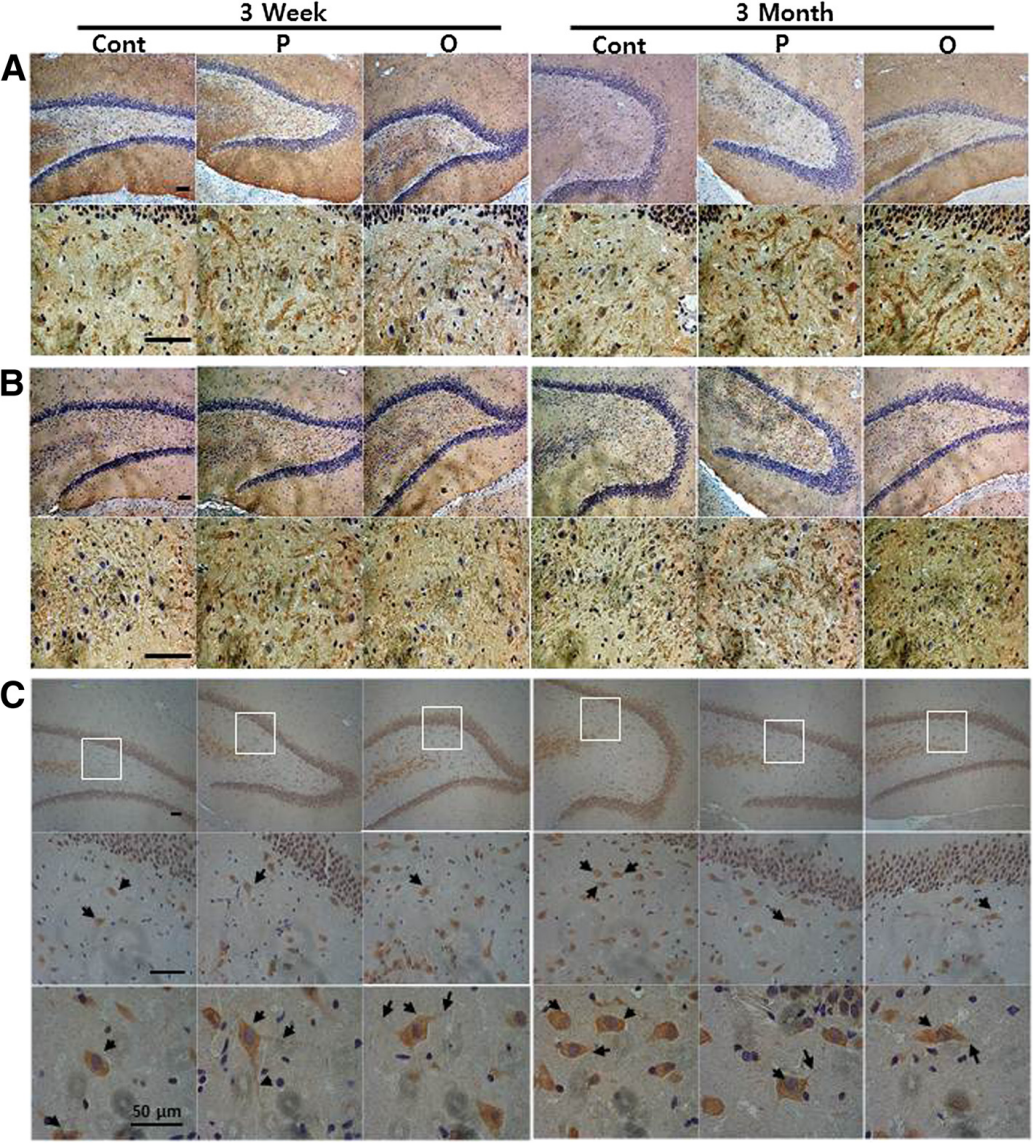
**Immunohistochemical analysis of the dentate gyrus of rats with improved cognitive function fed perilla diets.** Representative DAPI-labeled sections containing the dentate gyrus of rats fed perilla (P) or perilla oil (O) diets showed increased immunoreactivities by antibodies against AMPA receptor (**A**) and α-synuclein (**B**) compared to control (Cont) diet group. Whereas no significant changes in the density of immunoreactivity was observed in the sections stained by anti-neurofilament antibody (**C**). However, the immunoreactive cells by anti-neurofilament antibody showed longer neurites (indicated with arrows) in the hilus of dentate gyrus of rats fed perilla- or perilla-oil diets. Scale bars in the first left column represent 100 μm and are the same for other sections. Otherwise, scale bar was designated with a size for higher magnification (last raw in C).

**Figure 5:**
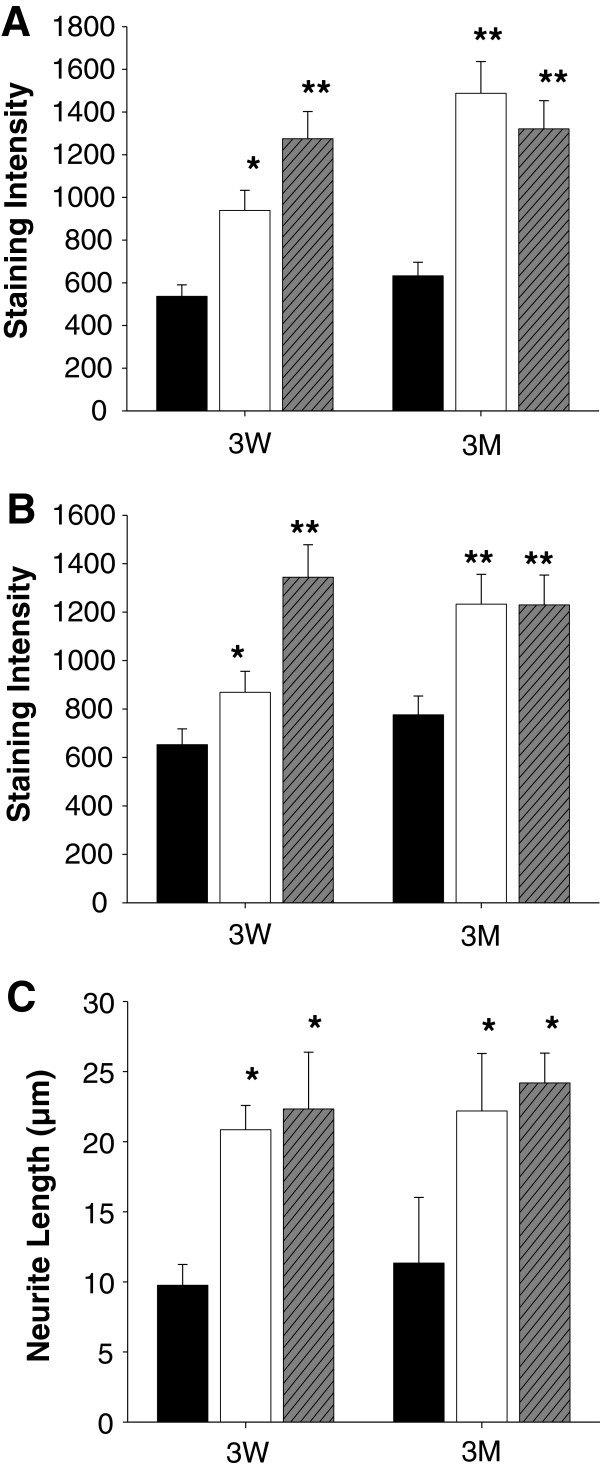
**Statistical presentation of the immunohistochemical staining of brain sections of rats.** Expression levels of AMPA receptor (**A**) and α-synuclein (**B**) represented by immunoreactivities in the sections were quantitated by a software, iSolution, and statistically compared (*, p<0.05, and **, p<0.01). The sections of brains of rats fed perilla (white bar) or perilla oil (grey bar) diet for 3 weeks (3W) or 3 months (3M) were more immunoreactive to the antibodies against AMPA receptor and α-synuclein than those of rats fed control (black bar) diet. Although anti-neurofilament antibody did not show a significant immunoreactivity (Figure 4C) between control and perilla groups in the sections, the cells stained by the anti-neurofilament antibody have significantly longer neurite projections (*, p<0.05, and **, p<0.01) (**C**).

As AMPA receptor, neurofilament and α-synuclein were all significantly up-regulated in the hippocampus of rats showing improved spatial learning and memory following perilla-diet intake, we questioned whether or not the up-regulation of protein expression had any histological effects on the hippocampus. To test this, we used primary antibody against AMPA receptor as a positive control for improved learning and memory function along with antibodies against α-synuclein, and neurofilament. We also performed immunohistochemistry staining on the sagittal microtome sections of rat brains (Figure 4). Tissue sections were prepared after completion of the behavioral studies. Anti-AMPA receptor antibody decorated the dentate gyrus of rats with significantly higher intensities following perilla-diet intake for 3 weeks or 3 months (Figure 5A) in comparison with control rats, suggesting that the rats indeed experienced improved spatial cognitive function by perilla-diets. Likewise, antibody against α-synuclein showed higher immunoreactivity in the dentate gyrus of ratsfed perilla- or perilla- oil-diet for 3 weeks or 3 months (Figure 5B). However, no significant differences were observed in the CA1 to 3 regions in the hippocampus or in the cortex (data not shown).

Contrary to the results of the proteomic (Table 1) and the Western blotting analyses (Figure 3) of the hippocampus, no significant differences in neurofilament M were detected between the two groups in terms of immunostaining intensitied (quantified as intensity of immunostained cells/mm^2^ dentate gyrus: control=12.0961; perilla=12.2860; perilla oil=12.2899, Figure 4C) in the dentate gyrus. However, cells stained by the anti-neurofilament M antibody showed different cell morphologies. Immunoreactive cells in the hilus of dentate gyrus of the control group showed less neurite projections compared to cells of the perilla-diet groups (Figure 4). To observe the projections, high magnification photomicrographs (representative images shown in (Figure 4C)) were taken, and the projections are indicated with arrows. The relative length of the projections measured as described in the Methods (Figure 5C) significantly increased in the perilla-diet groups, suggesting enhanced neuron projections in the dentate gyrus upon supplementation of perilla- or perilla oil-diet.

## Discussion

As synapses are labile and plastic structures, any micro-environmental changes in the brain can alter the number and structural configurations of existing synapses. Changes in neurological signaling by alterations of the gene expression profiles and lipid composition in the synaptic membrane can modulate cognitive function in the brain [18,19]. Thus, the goals of this study were to analyze proteomic changes in the brain upon supplementation of α-linolenic acid, an n-3 fatty acid, rich perilla, or perilla oil as well as to identify proteins closely related to the cognitive function.

Short term (for 3 weeks) feeding of 18:3n-3 fatty acid-rich perilla-diets to rats significantly improved spatial learning memory (Figure 1), changed the level of 22:6n-3 fatty acid, DHA, in the brain (Figure 2 and Additional file 1), and altered proteome profiles compared to the corn-oil based control diet group (Tables 1 and 2). These changes were also observed in the rat groups fed perilla-diets long term (for 3 months). From the proteomic analysis, we found that the differentially expressed proteins in the hippocampus of rats fed perilla-diets for 3 weeks were mainly annotated into several functional groups, including cytoskeleton and transport, whereas the major functional groups of those proteins from the 3 month groups were energy metabolism, neuron projection and apoptosis in addition to transport and cytoskeleton (Table 3). The annotation grouping in this study shows that the changes in fatty acid composition in the membrane of the brain of rats fed n-3 fatty acid rich perilla-diets altered the expression profiles of hippocampal proteins, including cytoskeleton and energy metabolism proteins, hereby affecting neurogenesis and synaptic plasticity. Further, changes in the protein expression profile grew more complex as the animals became older, suggesting that there are common and specific pathways for cognition in response to various stimuli, and the pathways involved in cognitive function were much more complicated for the mature brain than for the young one. Previously, we demonstrated that estrogen depravation in rats by ovariectomy results in alleviated spatial learning and memory in a T-maze test, and we observed significant proteomic changes in energy metabolism, cytoskeleton, and anti-oxidation [20]. These results indicate that cytoskeleton as a common ground in conjunction with other proteins from different functional groups, improves cognition through various changes in synaptic plasticity.

To understand how the differentially expressed proteins are involved in synaptic plasticity, and based on the fold differences in 2-D gel electrophoresis, we selected four proteins for verification and functional analysis of the proteomic data. Antibodies against AMPA receptor, neurofilament, a-synuclein, and β-soluble NSF attachment protein were used for Western blotting and immunohistochemistry analysis.

Firstly, Western blotting analysis using antibodies against the selected proteins were performed (Figure 3). Although AMPA receptor could not be detected by 2-D gel electrophoresis and its differential expression in the hippocampus was not clear, it was selected as a positive control since the receptor subunits are essential for long term potentiation induction and maintenance [16] and play important roles in spatial learning and memory [15]. Using anti-AMPA antibody, rats fed perilla-diets for 3 weeks or 3 months showed elevated AMPA receptor expression in the hippocampus, indicating that, together with the results from the T-maze test, n-3 fatty acid rich perilla-diets improved cognitive functions. Among the selected differentially expressed proteins in Tables 1 and 2, α-synuclein and neurofilament showed significantly increased expression, whereas β-soluble NFS attachment protein underwent slight changes in expression between the control group and perilla-diet groups in Western blotting analysis (Figure 3B). Further, the up-regulated proteins in the 2-D gel analysis showed higher expression in the Western blotting analysis. Interestingly, the perilla-diet group always showed lower protein expression than the perilla oil-diet group. This could be due to the higher availability of n-3 fatty acids in the digestive tract in the perilla oil group compared to perilla group (Figure 2). Oils in perilla seeds should be less available to the digestive enzyme system and thus less miscible and less absorptive in the intestine compared to the extracted perilla oil.

Secondly, using brain sections and antibodies whose target proteins were the most differentially expressed in the Western blotting analysis, we performed immunohistochemistry to examine where the proteins were expressed in the hippocampus as well as any changes in cell morphology. In addition, the specificity and reactivity of the available antibodies to the paraffin sections were considered.

α-Synuclein gene, which encodes a 140-amino-acid protein, is a key player in neurodegenerative diseases. The fibrillar β-sheet aggregation of α-synuclein is the major constituents of Lewy bodies and Lewy neurites in the pathogenesis of Parkinson disease [21]. However, in comparison to its identified roles in neuropathogenesis, the normal physiological function of α-synuclein in neurogenesis is not well understood. As a presynaptic protein, α-synuclein, is located in the membrane, interacts with tubulins, is enriched in presynaptic termini, and is involved in microtubule transport and neurotransmitter release [22-24]. Mice lacking α-synuclein gene show normal long-term potentiation (LPT) of glutamatergic synapses in the hippocampus, suggesting that α-synuclein is not essential for LTP in the hippocampus [22]. Nevertherless, the modulation of α-synuclein gene expression in transgenic mice alters hippocampal neurogenesis and synaptic plasticity in the dentate gyrus, suggesting that the protein might have a relationship with learning and memory [25,26]. Interestingly, it was reported that an increasing concentration of DHA in the membrane of the brain induces aggregation and chemical modification of α-synuclein *in vitro*[27]. Regarding the fact that the perilla-diet groups showed elevated DHA levels in the brain (Figure 2 and Additional file 1), n-3 fatty acid converted to DHA could modulate the redistribution of α-synuclein in the hippocampal presynapse for synaptic plasticity and improved cognitive function. This result supports the previous finding that the mRNA level of α-synuclein was elevated upon n-3 fatty acid treatment to rats for 3 weeks [9].

Neurofilament medium molecular weight protein (NF-M) is a neuron-specific intermediate filament protein that heteropolymerizes with other immunologically distinct neurofilaments, NF-L and NF-H, and constitutes a major part of the neuronal cytoskeleton [28]. The three different NF proteins form filaments at different stoichiometries under different physiological conditions, and the resulting sidearm structures of the C-termini of the NF proteins along the core fiber can interact with different cytoskeletal components to establish axonal cytoarchitecture [29]. Structures of the sidearms, which can be altered by NF stoichiometry, influence axonal transport and determines axonal caliber, which affects the conduction velocity of neurons. Therefore, alteration in the NF stoichiometry can contribute to neurodegenerative diseases [30] as well as other neurological functions. Up-regulated NF-M protein in the hippocampus of rats fed perilla-diets (Figure 3) can alter the neuronal cell morphology in the hilus of dentate gyrus, a neurogenic area (Figure 4C and 5C), thereby facilitating the conduction velocity of neurons to improve learning and memory. Moreover, the up-regulation of neurofilaments in the hilus of dentate gyrus facilitated neurite outgrowth in the perilla-diet groups, suggesting enhanced neurogenesis associated with improved spatial memory performance [31].

## Conclusion

This study shows that improved spatial learning and memory upon supplementation of n-3 fatty acid-rich perilla-diets is associated with differential expression of proteins in the hippocampus of rats. The proteins can be grouped into several functions, including cytoskeleton, energy metabolism, transport, neurogenesis, and apoptosis. Functional validation of the proteomic changes by immunohistochemical study using antibodies against neurofilament and α-synuclein showed differential immunoreactivities in the hilus of dentate gyrus, a neurogenesis center of the brain. These results indicate that improved cognitive function by supplementation with dietary α-linolenic acid from perilla induces changes in membrane fatty acid composition, especially, DHA, as well as in the immunoreactivity in a neurogenesis region of the hippocampus. Together, these changes culminate in alteration of synaptic plasticity.

## Methods

### Animals

Thirty 4-week old Sprague–Dawley male rats (Hyochang Science, Daegu, Korea), weighing 130–150 g were housed at 23°C and 60% humidity with *ad libitum* access to food and water under a 12/12-h light/dark cycle. After 1 week, the rats were divided randomly and equally into three groups and fed diets adjusted with the lipid contents of 7% with corn oil, perilla oil and powdered perilla seed, respectively, based on the AIN-93G formulation (Additional file 3). The rats were grown for 3 weeks or 3 months until the behavioral tests and brain proteome analyses. All animal procedures were in compliance with guidelines of Kyungpook National University (KNU) Animal Care and Use Committee.

### Behavioral test

The T-maze was constructed according to the measurements provided by Gerlai [32]. After a variable inter-trial interval in the goal box (15 sec on average), each rat was placed in the start box for the next trial. The pre-training session continued until the rat made its first correct avoidance response. For 2 weeks, the rats were trained until they made five correct avoidance responses in six consecutive training trials (acquisition session). On the last day of the second week, the actual test was conducted, after which a reversal session was performed with the correct goal box located opposite to the one used during acquisition. The measures included the latency to reach the correct goal box and the number of trials prior to correct avoidance.

### Protein sample preparation and 2-DE

Brain tissues from 7 individual brains per group were extracted after perfusion with ice-cold PBS and immediately stored at −80°C. For protein preparation, tissues were ground in liquid nitrogen and homogenized in lysis buffer containing 7 M urea, 2 M thiourea, 4% CHAPS, 1 M DTT and a mixture of protease inhibitors (Complete-Mini EDTA-Free, Roche, Indianapolis, USA), followed by sonication 20 times. The protein samples in lysis buffer were directly applied to immobilized pH gradient (IPG) strips (pH 3–10 and 4–7, 17 cm; Bio-Rad, Herculus, CA USA), and then separated on second dimensional SDS-PAGE. The gels were stained using a CBB R-250 method for visualization. The protein profiles on the gels were digitized by reflector mode scanning using an Epson Perfection V700 scanner (Nagano, Japan). Image analysis software, PDQuest v7.3 (BioRad), was used to compare the images and detect protein spots whose expression levels were significantly increased or decreased compared to control.

### Mass spectrometry analysis, protein identification, and bioinformatics

Protein spots of interest on gels were manually excised, washed with deionized water, and destained using 50 mM ammonium bicarbonate/acetonitrile (6:4, v/v) with vigorous shaking. Digestion was carried out by adding sequencing grade modified trypsin (Promega, Madison, WI, USA) onto the dried gel pieces, followed by incubation overnight at 37°C. The peptides were then extracted with extraction buffer (60% ACN in water and 0.1% TFA), and dried with the aid of a vacuum drier. From the concentrated peptide extract, 1 μl was taken and mixed with 1 μl of matrix solution (10% CHCA in 50% methanol and 0.1% TFA containing external standards such as bradykinin, angiotensin and neurotensin) on a target MALDI plate. The acquired mass spectra were analyzed by Mascot from Matrix Science ( http://www.matrixscience.com) and MS-Fit from Protein Prospector ( http://prospector.ucsf.edu/). Functional analysis of the differentially expressed proteins was carried out by searching the databases using the Uniprot KB ( http://www.ebi.ac.uk/uniprot/) searching tool, and the identified proteins were functionally annotated using an annotation search engine, DAVID Bioinformatics Resources 6.7 (National Institute of Allergy and Infectious Diseases (NIAID), NIH http://david.abcc.ncifcrf.gov/), an integrated knowledge base and analytic tools.

### Immunological analysis and staining

Proteins extracted from brain tissues were separated by SDS-PAGE and transferred to a PVDF membrane (Millipore, Billerica, MA, USA) for Western blotting analysis. After blocking the PVDF membrane with BSA, specific monoclonal primary antibodies (Cell Signaling, Danvers, MA, USA) against the differentially expressed proteins selected from the image analysis were properly diluted in 3% BSA with 0.1% Tween 20 in PBS and incubated with the membrane for 4 hr at 4°C. Following this, anti-mouse secondary antibody conjugated to horse radish peroxidase (Sigma-Aldrich, St. Louis, MO, USA) was diluted in 0.1% Tween 20 in PBS and incubated with the membrane for 1 hr at RT. Decorated bands were scanned using the LAS-3000 scanner (Fujifilm, Tokyo, Japan) after extensive washing with 0.1% Tween 20 in PBS and chemiluminescence staining (SuperSignal Pico-chemiluminescent Substrate, Pierce, IN).

Immunohistochemical staining of serial sagittal sections (4 μm) of paraffin-embedded brain tissues was performed using a Vectastatin ABC kit with the biotin/avidin/peroxidase system, including 3,3’-diaminobenzidine (DAB) as a chromogen and Hematoxylin as a counter stain (Vector, Burlingame, CA) by following the suggested manufacturer’s protocol. Specifically, paraformaldehyde-fixed and paraffin-embedded slide-mount tissue sections using 3 brains from each group were heated in boiling citrate-based antigen unmasking solution for 1 min for better antigen retrieval by the primary antibodies. Section slices were then incubated overnight with primary antibody in PBS containing 2% BSA and 2% normal goat serum followed by secondary antibodies coupled to horse radish peroxidase. The numbers of stained cells in the 4 well stained high magnification fields (400x), which were taken by using a H550L microscope and DS-Fi1c digital camera (Nikon, Tokyo, Japan), were counted using i-Solution (ver. 7.5; IMT i-Solution, Coquitlam, BC, Canada), an image analysis software.

Sections stained with anti-neurofilament antibody were used to measure the length of neurite projections using ImageJ software (NIH). Length data were statistically compared by Student’s *t*-test.

### Composition and concentration of fatty acids in the brain and perilla

Fatty acid analyses in the brain, perilla seeds, and perilla oil were performed on the samples (100 mg wet weight) treated with 4 ml of 0.5 M methanolic sodium hydroxide, and heated at 80°C for 5 min. After cooling, samples were methylated by addition of 3 ml of boron trifluoride in methanol and heated in an 80°C water bath for 5 min. The sample tube was then allowed to cool, after which 2 ml of hexane and 8 ml of distilled water were added. After vortexing for 1 min, the hexane fraction was removed from the tube for gas chromatographic analysis. Fatty acid methyl esters (FAMEs) of lipids from the samples were analyzed using an ACME 6100 gas chromatography system (YL Instrument, Anyang, Korea) equipped with a column (30 m in length, I.D. 0.32 mm wide bore, film thickness of 0.25 μm (J&W Scientific, Folsom, CA)). Fatty acid peaks were determined based on the retention times of the FAME standards. The area of externally added 17:0 standard was used to calculate the concentration of fatty acids by proportional comparison of the peak areas. All samples were run five times, and the values were averaged for all subsequent analyses.

## Competing interests

The authors declare that they have no competing interests.

## Authors’ contributions

JL and SP contributed to the overall conception and design of the project, carried out all the experiments, analysis and interpretation of the data, performed mass spectrometry analysis, and preparation of the manuscript. JYL carried out animal behavioral tests, mass spectrometric analysis and interpreting mass spectrometry data. YKY contributed with critical discussions on fatty acid analysis, fatty acid metabolism in brain and data interpretation in fatty acids. JSK contributed to the animal diet formulation, design of animal behavioral test, data analysis and interpretation. The corresponding author JL conceived the study, and participated in its design and coordination, data analysis and interpretation, and preparation of the manuscript. All authors read and approved the final manuscript.

## Supplementary Material

Additional file 1 **Table S1.** Fatty acid composition in the brain of rats fed control or perilla diets for 3 weeks or 3 months. Click here for file

Additional file 2 **Table S2.** 20 major proteins in the brain of rats fed perilla diets. Click here for file

Additional file 3 **Table S3.** Nutrient compositions of the diets. Click here for file
