# Supplementary material for: Improved spatial learning and memory by perilla diet is correlated with immunoreactivities to neurofilament and α-synuclein in hilus of dentate gyrus
Source: Proteome Sci. 2012 Dec 5;10:72. doi: 10.1186/1477-5956-10-72 (PMC3539918; doi:10.1186/1477-5956-10-72)
Supplement: Additional file 1 — Table S1. Fatty acid composition in the brain of rats fed control or perilla diets for 3 weeks or 3 months. [file 1477-5956-10-72-S1.docx]

**Table s1.** Fatty acid composition in the brain of rats fed control or perilla diets for 3 weeks or 3 months

| Fatty acid |  | 3 Week |  |  |  | 3 Month |  |
| --- | --- | --- | --- | --- | --- | --- | --- |
|  | Control | Perilla Seed | Perilla Oil |  | Control | Perilla Seed | Perilla Oil |
| C16:0 | 28.79±1.43 | 26.61±1.33 | 25.10±1.25 |  | 24.87±1.24 | 26.84±1.34 | 26.74±1.33 |
| C18:0 | 32.05±1.60 | 30.27±1.51 | 31.14±1.55 |  | 32.81±1.64 | 29.07±1.45 | 29.60±1.48 |
| C18:1 | 18.78±0.93 | 20.24±1.01 | 20.50±1.02 |  | 21.58±1.07 | 22.64±1.13 | 21.25±1.06 |
| C18:2n-6 | 0.89±0.04 | 0.80±0.04 | 0.87±0.02 |  | 1.04±0.05 | 0.88±0.04 | 0.86±0.04 |
| C20:3 | 0.00±0.00 | 0.58±0.02 | 0.52±0.02 |  | 0.00±0.00 | 0.55±0.02 | 0.47±0.02 |
| C20:4 | 9.53±0.47 | 9.38±0.46 | 9.42±0.47 |  | 9.58±0.48 | 8.01±0.40 | 8.14±0.40 |
| C22:6 | 9.93±0.49 | 12.09±0.60 | 12.42±0.62 |  | 10.09±0.50 | 11.97±0.59 | 12.90±0.64 |
