# Supplementary material for: Improved spatial learning and memory by perilla diet is correlated with immunoreactivities to neurofilament and α-synuclein in hilus of dentate gyrus
Source: Proteome Sci. 2012 Dec 5;10:72. doi: 10.1186/1477-5956-10-72 (PMC3539918; doi:10.1186/1477-5956-10-72)
Supplement: Additional file 2 — Table S2. 20 major proteins in the brain of rats fed perilla diets. [file 1477-5956-10-72-S2.docx]

**Table s2.** 20 major proteins in the brain of rats fed perilla diets

| No. | MOWSE Score | Coverage % | Accession # | Protein Name |
| --- | --- | --- | --- | --- |
| 1 | 2.71E+08 | 32.7 | P19527 | Neurofilament light polypeptide |
| 2 | 34601 | 9.8 | Q66HD0 | Endoplasmin |
| 3 | 4.94E+06 | 16.3 | O08838 | Amphiphysin |
| 4 | 118836 | 12.3 | P12839 | Neurofilament medium polypeptide |
| 5 | 1.21E+07 | 23.3 | Q6P9V9 M | Tubulin alpha-1B chain |
| 6 | 1487 | 13.8 | P0C2N6 | Histone-lysine N-methyltransferase SUV420H2 |
| 7 | 1.08E+11 | 54.9 | P62258 M | 14-3-3 protein epsilon |
| 8 | 1.56E+11 | 35 | P47942 M | Dihydropyrimidinase-related protein 2 |
| 9 | 3.86E+15 | 51.6 | P04764 | Alpha-enolase |
| 10 | 357723 | 26.2 | P62879 M | Guanine nucleotide-binding protein G(I)/G(S)/G(T) subunit beta-2 |
| 11 | 32027 | 7.5 | Q5XIS9 | Serine/threonine-protein kinase D2 |
| 12 | 4851 | 12.9 | P07335 | Creatine kinase B-type |
| 13 | 22533 | 8.9 | O54853 | Potassium voltage-gated channel subfamily H member 6 |
| 14 | 8.46E+17 | 43 | P63018 M | Heat shock cognate 71 kDa protein |
| 15 | 86384 | 16.4 | P63039 M | 60 kDa heat shock protein, mitochondrial |
| 16 | 5.36E+06 | 19.8 | P23565 | Alpha-internexin |
| 17 | 9.37E+13 | 40.7 | P47819 | Glial fibrillary acidic protein |
| 18 | 74377 | 27.3 | P35704 | Peroxiredoxin-2 |
| 19 | 3.42E+06 | 32.3 | O88989 | Malate dehydrogenase, cytoplasmic |
| 20 | 8.43E+08 | 33.2 | P42123 | L-lactate dehydrogenase B chain |
