# Supplementary material for: Improved spatial learning and memory by perilla diet is correlated with immunoreactivities to neurofilament and α-synuclein in hilus of dentate gyrus
Source: Proteome Sci. 2012 Dec 5;10:72. doi: 10.1186/1477-5956-10-72 (PMC3539918; doi:10.1186/1477-5956-10-72)
Supplement: Additional file 3 — Table S3. Nutrient compositions of the diets. [file 1477-5956-10-72-S3.docx]

| **Ingredients** | **Composition^*^** | | |
| --- | --- | --- | --- |
|  | **AIN93G** | **Perilla-diet** | **Perilla Oil-diet** |
| Casein | 200 | 160 | 200 |
| Corn Starch | 397.486 | 332.486 | 397.486 |
| Dextrose | 132 | 132 | 132 |
| Sucrose | 100 | 100 | 100 |
| Cellulose | 50 | 10 | 50 |
| **Soybean Oil** | **70** | **0** | **0** |
| **Powdered Perilla** | **0** | **215^a^** | **0** |
| **Perilla Oil** | **0** | **0** | **70** |
| t-BHQ | 0.014 | 0.014 | 0.014 |
| Mineral Mix | 35 | 35 | 35 |
| Vitamine Mix | 10 | 10 | 10 |
| L-cystein | 3 | 3 | 3 |
| Choline Bitratarate | 2.5 | 2.5 | 2.5 |
| **Total** | **1000** | **1000** | **1000** |

**Table s3.** Nutrient compositions of the diets

*All values are on a dry matter basis, except powdered perilla, which contains 36% water..

a.The energy value for the Perilla-diet was adjusted based on the formulation of AIN93G by reducing the amount of carbohydrate content (corn starch) measured in perilla seeed.
